# Genome-wide binding of the orphan nuclear receptor TR4 suggests its general role in fundamental biological processes

**DOI:** 10.1186/1471-2164-11-689

**Published:** 2010-12-02

**Authors:** Henriette O'Geen, Yu-Hsuan Lin, Xiaoqin Xu, Lorigail Echipare, Vitalina M Komashko, Daniel He, Seth Frietze, Osamu Tanabe, Lihong Shi, Maureen A Sartor, James D Engel, Peggy J Farnham

**Affiliations:** 1 Genome Center, University of California at Davis, Davis, CA 95616, USA; 2 Department of Cell and Developmental Biology, University of Michigan Medical School, Ann Arbor, MI 48109, USA; 3 Center for Computational Medicine and Bioinformatics, University of Michigan Medical School, Ann Arbor, MI 48109, USA

## Abstract

**Background:**

The orphan nuclear receptor TR4 (human testicular receptor 4 or NR2C2) plays a pivotal role in a variety of biological and metabolic processes. With no known ligand and few known target genes, the mode of TR4 function was unclear.

**Results:**

We report the first genome-wide identification and characterization of TR4 *in vivo *binding. Using chromatin immunoprecipitation followed by high throughput sequencing (ChIP-seq), we identified TR4 binding sites in 4 different human cell types and found that the majority of target genes were shared among different cells. TR4 target genes are involved in fundamental biological processes such as RNA metabolism and protein translation. In addition, we found that a subset of TR4 target genes exerts cell-type specific functions. Analysis of the TR4 binding sites revealed that less than 30% of the peaks from any of the cell types contained the DR1 motif previously derived from *in vitro *studies, suggesting that TR4 may be recruited to the genome via interaction with other proteins. A bioinformatics analysis of the TR4 binding sites predicted a *cis *regulatory module involving TR4 and ETS transcription factors. To test this prediction, we performed ChIP-seq for the ETS factor ELK4 and found that 30% of TR4 binding sites were also bound by ELK4. Motif analysis of the sites bound by both factors revealed a lack of the DR1 element, suggesting that TR4 binding at a subset of sites is facilitated through the ETS transcription factor ELK4. Further studies will be required to investigate the functional interdependence of these two factors.

**Conclusions:**

Our data suggest that TR4 plays a pivotal role in fundamental biological processes across different cell types. In addition, the identification of cell type specific TR4 binding sites enables future studies of the pathways underlying TR4 action and its possible role in metabolic diseases.

## Background

There are an estimated 1400 site-specific DNA binding factors encoded in the human genome [1]. Although these factors can influence transcription when their binding sites are cloned in front of core promoters, they usually do not function alone. Most often, individual transcription factors collaborate to orchestrate gene expression through combinatorial binding to regulatory regions in chromatin [2]. These regions, termed *cis *modules, thereby activate, repress or otherwise epigenetically modify the transcriptional responses of individual genes. Elucidating the position and activities of individual *cis *modules using reporter genes is time consuming and expensive. With recent advances in DNA sequencing technology, it is now feasible to generate global protein-DNA interaction profiles by chromatin immunoprecipitation (ChIP) followed by ultra-high-throughput sequencing [3]. *Cis *modules can then often be identified by applying bioinformatics searches for one or more *cis *motifs recognized by unrelated alternative factors near the binding sites of the factor analyzed by ChIP-seq or by the co-localization of bound sites for two or more unrelated different site-specific factors.

Nuclear receptors (NRs) represent a special class of transcription factors that direct target gene transcription in a ligand-dependent fashion. NRs contain a DNA-binding domain that recognizes a specific DNA sequence, as well as a ligand binding domain that renders these factors environmentally-dependent regulators via interaction with distinct cognate ligands [4]. The great majority of NRs homodimerize or heterodimerize with another NR, and then bind to two copies of a repeated hexanucleotide sequence (called a half-site) separated by variable spacing [5]. The half-site consensus, AGGTCA, can occur in either orientation and variation from the consensus allows numerous alternative binding sites of (probably) variable affinity [5]. Based on the number of spacer nucleotides separating the two half-sites and the orientation of the two half-sites relative to each other, NR binding sites have been categorized as direct repeats (DR0 - DR8), everted repeats (ER0 - ER8) or inverted repeats (IR0-IR8) [5].

NR2C2 (human testicular receptor 4, TR4, in the older nomenclature) belongs to the nuclear receptor superfamily and is termed an orphan receptor due to the fact that no ligand has been discovered [6-8]. TR4 was initially identified in hypothalamus, prostate, and testis cDNA libraries, but has since been demonstrated to be broadly expressed in many physiological systems [9,10]. For example, TR4 has been shown to activate target gene expression in liver carcinoma HepG2 cells [11]. In contrast, in erythroid cells, TR4 can heterodimerize with another closely related family member (TR2, or NR2C1) and binds to a DR1 (direct repeats with one nucleotide spacer) element to repress target gene transcription [12-15]. The binding affinity of the TR4 homodimer for the DR1 element *in vitro *is equivalent to that of the TR2:TR4 heterodimer [15], and TR4 mRNA is more abundant than TR2 in human erythroid cells (Tanabe, unpublished observations). However, the broader physiological functions for, and the *in vivo *genome-wide binding patterns of, this broadly expressed nuclear receptor are obscure. We therefore chose to initially investigate genome wide TR4 binding anticipating that these studies might reveal some common, but also perhaps some tissue-specific, metabolic processes to which this factor contributes.

In this study we investigated the first genome-wide identification of cellular targets of TR4 and preliminary characterization of TR4 *in vivo *binding in multiple cell types, including those in which TR4 has been suggested to be an activator (liver) and cells in which TR4 has been suggested to be a repressor (blood). Using ChIP-seq, we determined TR4 *in vivo *binding in four human ENCODE cell lines: K562 erythroleukemia cells, HepG2 liver carcinoma, HeLa cervical carcinoma, and GM12878 immortalized lymphoblast cells. TR4 binding patterns identified in the four diverse cell lines suggest that this factor controls cell metabolism by binding to the proximal promoter regions that are common to several hundred genes. Motif analysis shows that TR4 strongly prefers a DR1 sequence to all other categories of repeat elements *in vivo*. By integration of TR4 binding data with histone modification patterns and other genomic structures, we predict, and then experimentally test, putative *cis *modules.

## Results and Discussion

### Identification of genome-wide TR4 binding sites

**Figure 1:**
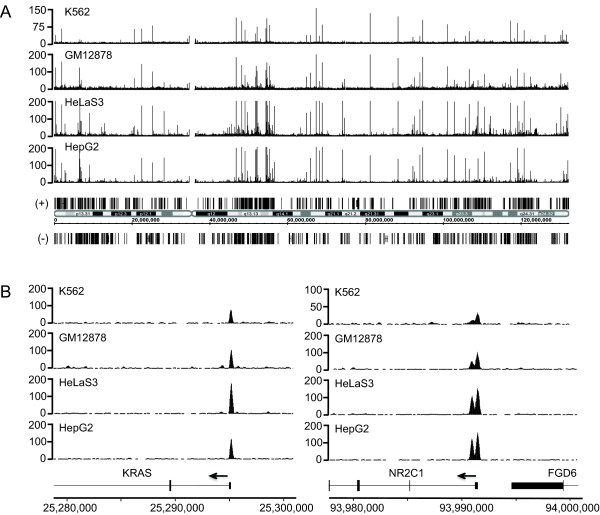
**Comparison of TR4 targets in 4 different cell types**. ChIP-seq binding patterns of TR4 (NR2C2) from K562, GM12878, HeLa, and HepG2 cells are shown (A) for entire chromosome 12 and (B) for target genes KRAS and TR2 (NR2C1). The number of tags reflecting the ChIP enrichments is plotted on the y axis and chromosomal coordinates (hg18) are shown on the x axis. RefSeq genes are indicated in (+) and (-) orientation. Target genes KRAS and TR2 are in (-) orientation as indicated by the arrows.

With no known ligand and few proposed binding sites in mouse and human cell lines [16-19], the function of the TR4 orphan nuclear receptor was largely unknown when we began these studies. Previous studies examined its function in different blood cells and found that TR4 bound to the CD36 promoter in macrophages [20] and to the GATA1 enhancer G1HE [12] in CD34^+ ^cells, but only after *in vitro *differentiation for 11 days. To further elucidate biological roles for TR4, we set out to identify *in vivo *TR4 binding sites throughout the entire human genome using chromatin immunoprecipitation followed by high throughput sequencing (ChIP-seq). We wanted to compare its binding profiles in cells derived from different tissue types. We chose to identify TR4 targets in cell types selected by the ENCODE Consortium (http://www.genome.gov/10005107), including human chronic myelogenous leukemia cells (K562), human cervical carcinoma cells (HeLa), lymphoblastoid cells (GM12878), and hepatocellular carcinoma cells (HepG2). By characterizing its binding in these cell lines, we could compare TR4 binding sites with other transcription factor binding sites and histone marks determined by other ENCODE groups examining these same cell types. We first validated the presence of TR4 protein in these cell lines by Western Blot analysis (see Additional file 1). We began our ChIP experiments using the hematopoietic cell line K562 and the liver cell line HepG2, but were unable to confirm TR4 enrichment at targets previously published in the specialized and differentiated hematopoietic cells. Therefore, we initially proceeded without having positive controls for the ChIP assays. We prepared sequencing libraries from ChIP experiments from two independently grown batches of HepG2 cells. Samples were sequenced using the Illumina GA2 platform and ChIP-seq data were analyzed using the Sole-search software (http://chipseq.genomecenter.ucdavis.edu/cgi-bin/chipseq.cgi; [21]). Only sequences that uniquely matched those in the human genome were retained for analysis. 9.7 million sequence reads were obtained from replicate 1 and 8.2 million from replicate 2. Using the Sole-search peak calling program with default settings (FDR 0.0001, alpha value 0.001), 1,547 and 2,246 TR4 binding sites were identified in HepG2 cells for replicate 1 and replicate 2, respectively. 1,243 (80%) of the 1,547 peaks called from replicate 1 were also present in the 2,246 peaks called from replicate 2. This overlap demonstrates good reproducibility between biological replicates. To obtain the final list of 2,672 TR4 binding sites in HepG2 cells, all reads (17.8 million) from both biological replicates were merged. We then performed TR4 ChIP experiments for the other cell types and used standard PCR to confirm enrichment at three sites (TNFIAP1, SCAP, ECSIT) previously identified in HepG2 cells (see Additional file 2 for primer information; see Additional file 3 for representative PCR validation). ChIP-seq libraries were then prepared from two biological replicates using the TR4 antibody resulting in 23 million sequence reads for HeLa cells, 30 million for GM12878 cells and 16 million for K562 cells (see Additional file 4 for a summary of the data analysis). 1,767 TR4 binding sites were identified in HeLa cells, 1,180 TR4 binding sites in GM12878 cells and 732 TR4 binding sites in K562 cells; see Figure 1 for the binding patterns of TR4 across the entire chromosome 12 in all four cell types.

**Figure 2:**
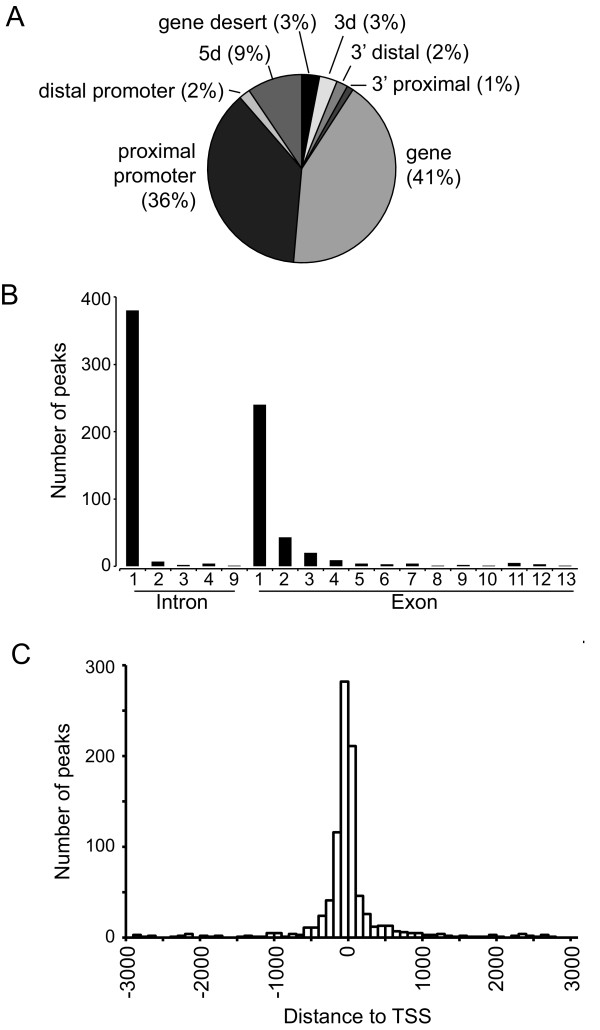
**Location analysis of TR4 binding sites in HeLa cells**. (A) Shown is a pie chart indicating the distribution of called TR4 peaks. Categories are based on the distance of the peak to the nearest RefSeq gene: 5 d (10 - 100 kb upstream of TSS), distal promoter (2 - 10 kb upstream of TSS), proximal promoter (<2 kb upstream of TSS), gene (exon or intron), 3' proximal (<2 kb downstream of the last exon), 3' distal (2 - 10 kb downstream of the last exon), 3 d (10 - 100 kb downstream of the last exon), and gene desert (>100 kb from a RefSeq gene). (B) Distribution of peaks found within genes. (C) Histogram showing the distribution of peak distances relative to the transcription start site (TSS) of the nearest gene. Peaks were combined in 100 bp bins

The position to which a transcription factor binds relative to the start site of transcription can provide insight into how the factor regulates transcription. For example, E2F family members bind to core promoter regions and are thought to stimulate transcription by interaction with the basal transcription machinery [22,23]. In contrast, other transcription factors, such as GATA1 or TCF4 (TCF7L2), show significant binding to sites often located more than 10 kb away from the gene that they regulate [21,24], suggesting that these factors may regulate transcription by looping mechanisms. Although the number of TR4 binding sites varied among the different cell types, location analysis revealed that TR4 preferentially binds close to the transcription start sites of its target genes. The majority of TR4 binding sites (65-82%) is located either in the proximal promoter (up to 2 kb upstream of TSS) or is found within the first exon or first intron of a RefSeq gene. In HeLa cells, 36% of TR4 binding occurred in the proximal promoter and 41% in the gene region, mainly in the first exon or first intron (Figure 2A and 2B). To further characterize TR4 binding sites, TR4 ChIP-seq reads were organized into 100 bp bins relative to the start site of transcription. The distribution of TR4 peaks relative to the transcription start site demonstrated that the majority of TR4 binding occurs between 1 kb upstream and 1 kb downstream of a TSS (Figure 2C). For example, 1,135 (63%) of the 1,767 HeLa binding sites were located within ± 1 kb from a TSS (see Additional file 4 for results from all cell types). This preference was also reflected in an elevated median height of peaks near a TSS; the median peak value was 114 for peaks within ± 1 kb of a TSS, but only 50 for peaks outside this range. For the rest of our studies, we therefore focused on the targets found within 1 kb of a TSS. This encompassed 1,154 TR4 binding sites for HeLa, 1,732 for HepG2, 537 for K562 and 535 for GM12878 cells.

**Figure 3:**
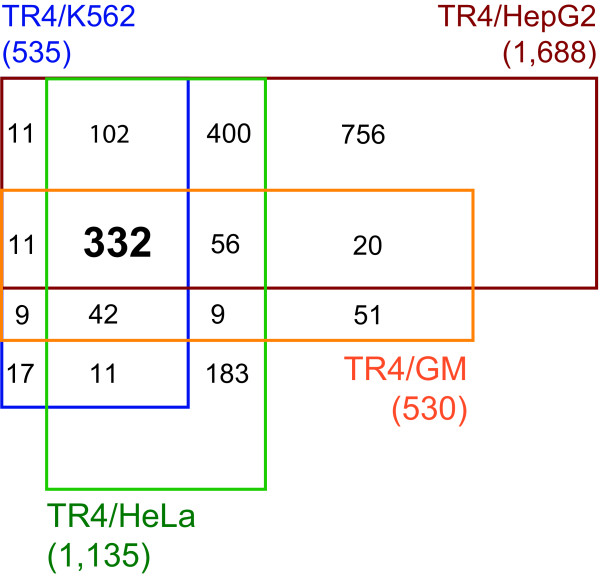
**Overlap of TR4 target genes in 4 cell types**. A target gene is defined as the nearest gene to a ChIP-seq peak. In some cases a target gene was contained more than one peak. Genome-wide TR4 ChIP-seq has identified 535 target genes in K562, 1,688 in HepG2, 1,135 in HeLa, and 530 in GM12878 cells within ± 1 kb of a transcription start site. 332 genes are identified as common targets in the 4 cell types.

A significant fraction of TR4 binding sites was shared among cell types (Figure 1B). For example, out of the 537 TR4 binding sites in K562 cells, 504 (94%) are also occupied in HeLa cells, 471 (88%) are also bound in HepG2 cells and 406 (76%) are also bound in GM12878 cells. When comparing 1,157 TR4 binding sites from HeLa with 1,732 from HepG2 cells, we found 922 (80%) were shared TR4 target sites. We next matched the TR4 peaks to the nearest gene. In some cases more than one peak matched to a given gene. As a consequence, the number of TR4 binding sites is slightly higher than the number of target genes. We compared 1,135 TR4 target genes from HeLa, 535 from K562, 530 from GM12878 and 1,688 from HepG2 cells (Figure 3). 532 target genes were shared in at least 3 cell types and 332 target genes were shared among all four cell types. While blood cells shared most of their TR4 targets, liver cells contained the largest number of unique target genes. TR4 may regulate genes important for basic biological processes shared in multiple cell types, while it may play an additional role in regulating cell type specific genes.

### TR4 target genes are involved in fundamental biological processes

**Figure 4:**
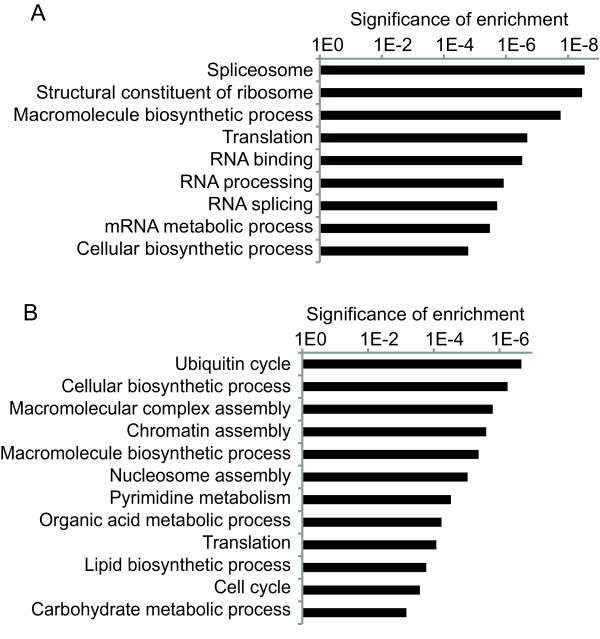
**Functional enrichment analysis of TR4 target genes**. (A) Targets common to all 4 cell types and (B) targets unique to HepG2 cells. Significantly enriched gene ontology terms for biological processes are shown on the y axis; the x axis represents p-values for each enriched category.

As shown above, the majority of TR4 targets are shared between different cell types. To shed light on the common function of genes targeted by TR4, gene ontology analysis was performed using ConceptGen (http://conceptgen.ncibi.org/core/conceptGen/index.jsp; [25]) to identify the functional categories enriched in the overlapping targets in 4 cell types (p-value < 0.05, modified Fisher's exact test). All Entrez Genes were used as background to determine the significance of over-representation. Categories of TR4 target genes are highly enriched in fundamental biological processes, such as RNA metabolism and protein translation (ribosome) (Figure 4A). In addition, TR4 may also regulate cell type-specific genes. To test this hypothesis, we performed gene ontology analysis on genes found in only one cell type. The number of unique target genes in K562, HeLa, and GM12878 cells was not sufficient to perform meaningful gene ontology analysis. However when 756 TR4 target genes specific to HepG2 cells were analyzed, we found some unique functional categories (Figure 4B). HepG2 specific target genes were significantly enriched for ubiquitin cycle, nucleosome, chromatin assembly and metabolic processes, particularly those involving organic acid, carbohydrates, and lipids. Interestingly, a few previous studies have suggested a role for TR4 in gluconeogenesis [16]. Furthermore, TR4 may exert its function by sensing lipids and the presence of fatty acids was found to enhance cofactor recruitment to TR4 [26] suggesting an important role for lipids in TR4 function.

**Figure 5:**
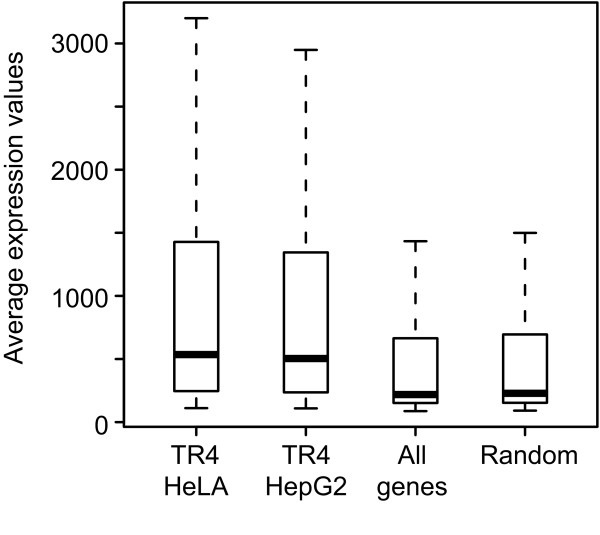
**Expression analysis of TR4 target genes**. Box-and-whisker diagrams show the range of expression values of TR4 bound genes in HeLa and HepG2 cells in comparison to expression values of all genes present on the HepG2 expression array and to the set of 3000 randomly selected genes. Expression values are plotted on the y axis. The central line in the box-and-whisker plots shows the position of the median, the upper and lower boundaries of the box represent the location of the upper (75th percentile) and the lower (25th percentile) quartiles, respectively. Data outliers are not shown.

In recent years it has become evident that transcription factors often play dual roles, affecting activation as well as repression of target genes. Previous studies have implicated TR4 in both activation and repression of cellular target genes [7]. TR4 binds to DNA as a homodimer, but preferentially forms heterodimers with the orphan receptor TR2 [27]. Recently, a global atlas for transcription factor networks has been assembled based on physical protein-protein interactions using mammalian two hybrid data [28]. This study identified TR4 (NR2C2), Nuclear Receptor Interacting Protein 1 NRIP1 (RIP140), and histone deacetylases HDAC 3 and HDAC4 as proteins interacting with TR2 (NR2C1). NRIP1 may function as a corepressor or coactivator depending on the interacting protein [29]. Furthermore, post translational modifications of TR4 influence its interaction with cofactors [30]. Phosphorylation of TR4 is accomplished by MAP kinases and results in recruitment of NRIP1. On the other hand, dephosphorylated TR4 recruits the coactivator pCAF. We wanted to determine whether TR4 target genes are expressed or silenced. For this purpose, we matched TR4 target genes in HeLa and HepG2 cells (1,135 and 1,688 respectively) to their RNA expression values from Illumina expression arrays (Figure 5). The median expression value of TR4 target genes in HeLa and HepG2 cells (median expression value 535 and 504, respectively) is higher than the median expression value of all genes from the HepG2 expression array (median expression value 219). TR4 target genes are also expressed at higher levels than a set of 3000 randomly selected genes from the HepG2 expression array (median expression value 228). Based on RNA expression analysis, TR4 target genes are generally expressed.

**Figure 6:**
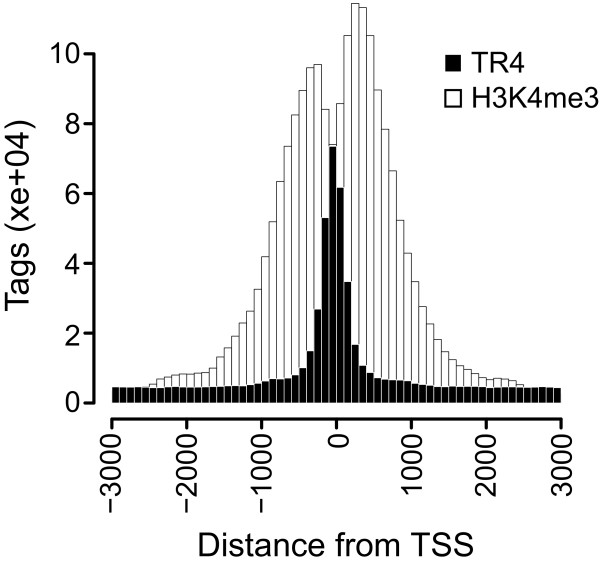
**TR4 binding relative to nucleosomes**. Positions of the histone mark H3K4me3 and TR4 occupancy are plotted for the 735 genes bound by TR4 in K562 cells. Sequence tags in bins of 100 base pairs are plotted on the y axis; distance to transcription start site is shown on the x axis.

The correlation between TR4 binding and expression of target genes suggests that TR4 binds to open accessible chromatin regions. To test this hypothesis, we examined the epigenetic signature at TR4 binding sites using ChIP-seq data of various histone marks in K562 cells. Overlap of TR4 binding sites with histone marks typical for open and repressed chromatin was determined using the gffOverlap tool from Sole-search (http://chipseq.genomecenter.ucdavis.edu/cgi-bin/chipseq.cgi; [21]). A distance of 200 base pairs between peaks was allowed to take nucleosome positioning into account. A remarkable 534 of the 537 TR4 target sites in K562 cells were also occupied by H3K4me3, which is a mark for accessible chromatin. No significant overlap with the repressive chromatin marks H3K27me3 or H3K9me3 was found (2 and 5 peaks, respectively). It has been shown in yeast and also human cells that transcription factors often bind in the linker region between nucleosomes [3,31]. To determine whether TR4 binding occurs in nucleosome depleted regions, we analyzed sequence tag density for TR4 and H3K4me3 binding relative to the transcription start sites (Figure 6). TR4 binding was highest within 100 base pairs upstream of the TSS while the histone mark H3K4me3 is lowest in this region and reaching maximum where TR4 binding tails off, suggesting predisposition of TR4 binding sites to the linker region.

### Motif analysis suggests the importance of ETS family members in TR4 action

**Figure 7:**
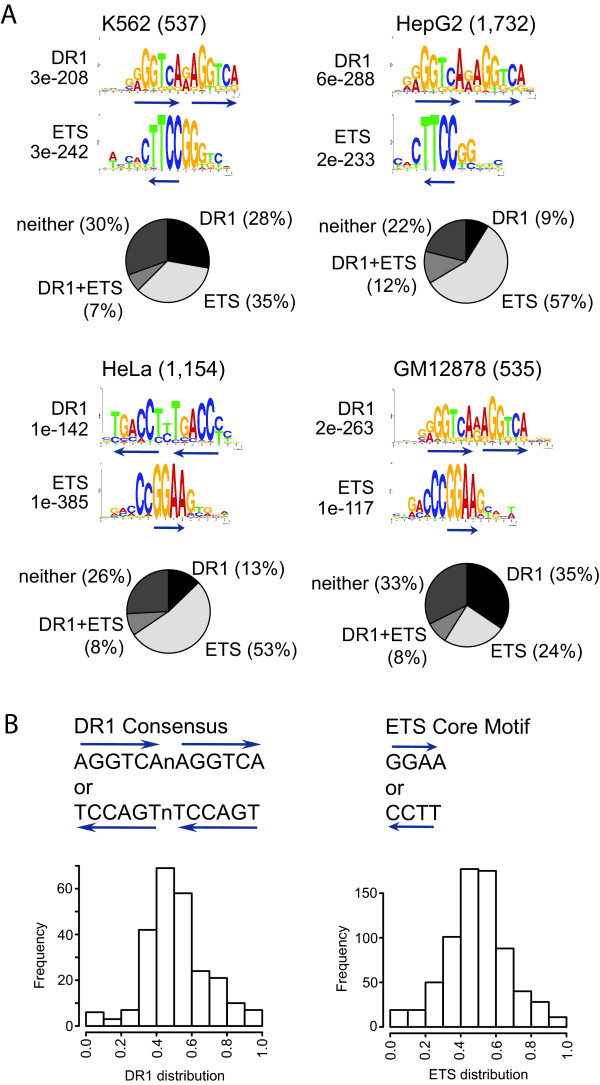
**Motif analysis of TR4 binding sites**. (A) Sequences for TR4 binding sites located within 1 kb upstream and downstream of a TSS were retrieved. Significantly overrepresented motifs within TR4 binding sites were identified by MEME. The number of targets is indicated in parenthesis. E-values indicate significance of a given motif. Pie charts show occurrence of DR1 alone, ETS alone, DR1 and ETS, and neither of these motifs within TR4 binding sites. (B) DR1 motif and ETS core motif are depicted in either orientation. Occurrence of DR1 and ETS motifs relative to TR4 peak center in HeLa cells is shown in a histogram. Peak frequency is plotted along the y axis; distance from the peak center is plotted on the x axis. Similar results were obtained with the other 3 cell types, histograms are not shown.

*In vitro *experiments have shown that TR4 binds to the direct repeat (DR) of AGGTCA, which is the consensus binding site for a number of nuclear hormone receptors including estrogen receptor alpha and PPAR. Further studies have indicated that TR4 can bind to direct repeats separated by zero to five nucleotides (DR0 - DR5) [11,13,17,32]. However, all previous studies were performed using *in vitro *assays. We used the *de novo *motif discovery program MEME to identify motifs overrepresented in TR4 binding sites to determine if TR4 has the same specificity *in vivo*. To allow identification of DR elements and its spacing and flanking nucleotides, the minimum motif length was set between 12 (length of two half sites with no spacing in between) and 20 nucleotides (length of two half sites with up to 8 nucleotides in between). The canonical DR motif with one nucleotide spacing (DR1) was significantly overrepresented in all four cell types with the preferred spacing nucleotide being an A or G (Figure 7A). The canonical DR1 motif accounts for about 150 TR4 binding sites (28% in K562, 9% in HepG2, 13% in HeLa, and 35% in GM12878 cells). Interestingly, the % of peaks having a DR1 motif is much higher in the blood cell lines (K562 and GM12878) than in the other two cell types. The lack of the DR1 motif in the remaining peaks may indicate that TR4 associates with some sites only indirectly by binding to a different transcription factor.

Transcription factors often regulate expression of nearby genes in combination with other transcription factors through complex *cis *regulatory modules [33]. Our initial motif analysis revealed the significant recurrence of an ETS motif in addition to the DR1 element. Members of the ETS transcription factor family such as ELK4, E74A, and GABPA recognize the ETS core motif GGAA. Using 13,010 human promoter sequences, the ETS motif has been identified as one of those motifs exhibiting statistically significant clustering near the transcription start site [34]. The ETS motif was predominantly found in the promoters of genes with essential cellular functions, such as ribosomal genes, mitochondrial ribosomal genes, basal transcription factor genes and proteosomal genes. The ETS motif is not only found at genes regulating similar processes as TR4 target genes, but also preferentially occurs 100 base pairs upstream of a transcription start site. The ETS motif occurs in a significant portion of TR4 binding sites (35% in K562, 57% in HepG2, 53% in HeLa, and 24% in GM12878 cells). Only about 10% of target genes contain both the DR1 and the ETS motif (Figure 7A). Combining both motifs can account for 67-78% of TR4 peaks (70% in K562, 78% in HepG2, 74% in HeLa, and 67% in GM12878 cells) suggesting a combinatorial role for ETS family members in TR4 function. Similar results were obtained using other *de novo *motif discovery programs such as NHR-Scan [5] and W-ChIPMotifs [35].

It has been postulated that the true binding site for transcription factors should be located under the center of the peak [36]. We analyzed the distribution of both motifs relative to the center of the TR4 binding sites and found that the DR1 as well as the ETS motif are located under the peak center (Figure 7B). The close proximity of these binding sites suggests a *cis *regulatory network involving TR4 and ETS family members.

### ETS transcription factor ELK4 co-occupies TR4 target sites

We wanted to test the hypothesis that TR4 and a member of the ETS family co-localize with TR4 *in vivo *using ChIP-seq. Motif analysis implicates the ETS family, but does not provide information as to which family member might bind to TR4 target sites. There is a high degree of functional redundancy between different members of the ETS transcription factors. Comparison of ELK1 and GABPA binding regions revealed redundant as well as unique targets between the two ETS family members [37,38]. It has also been shown that ETS transcription factors interact with other transcription factors to regulate gene expression. For example, ELK1 is thought to function through cooperation with the serum response factor SRF [37,39]. ChIP-chip analysis showed that 22% of all ELK1 binding regions were also bound by SRF, while the majority of ELK1 targets is SRF-independent.

**Figure 8:**
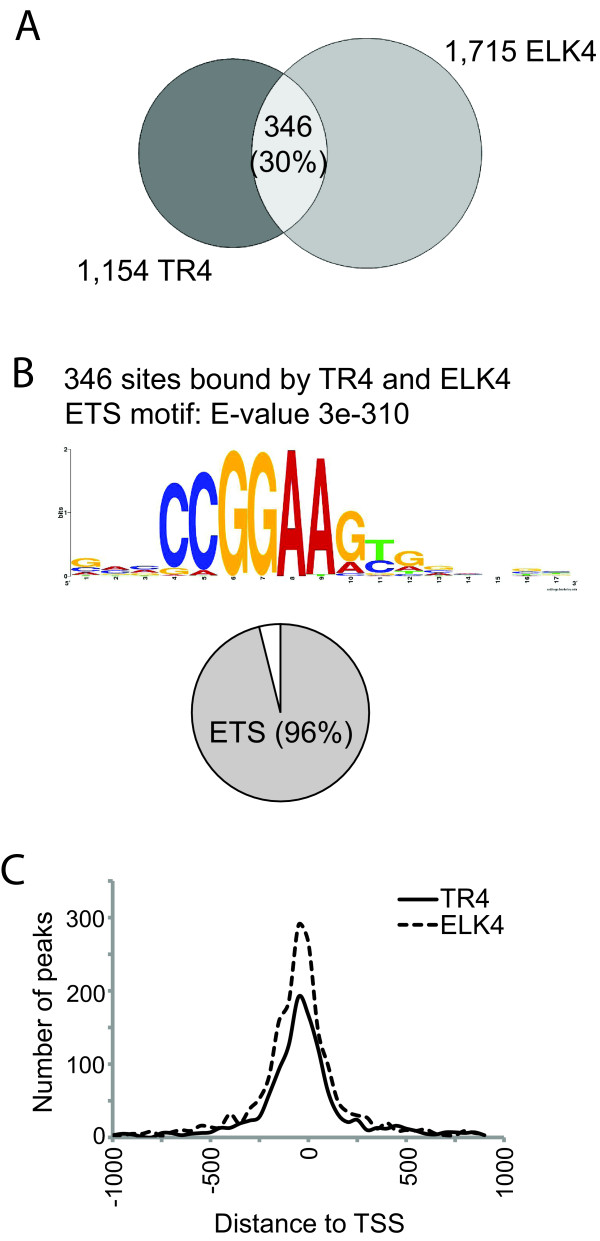
**Overlap of TR4 and ELK4 binding sites in HeLa cells**. (A) Venn diagram shows the overlap of TR4 and ELK4 binding sites within ± 1 kb of transcription start site. (B) Motif analysis was performed on the 346 sites bound by both factors; the overrepresented ETS motif is shown. Pie chart shows the occurrence of the DR1 motif, ETS motif and neither of these motifs. (C) Histogram shows binding of TR4 and ELK4 relative to the transcription start sites. Binding sites were binned into 50 base pair bins. Number of peaks is shown on the y axis; distance relative to transcription start site is plotted on the x axis.

**Figure 9:**
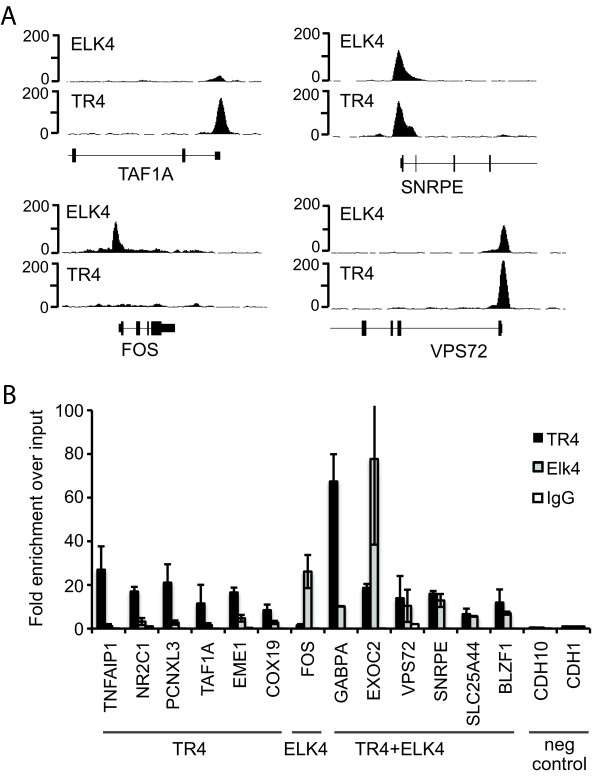
**TR4 and ELK4 bind to common target genes**. (A) ChIP-seq signal track of TR4 and ELK4 enrichment at common and unique target sites in HeLa cells. TAF1A promoter region is bound by TR4 only; C-FOS promoter region is occupied by ELK4 only, while EXOC2, SNRPE and VPS72 gene promoters are occupied by TR4 and ELK4. Number of sequence tags representing enrichment is plotted on the y axis. (B) ChIP validation of TR4 and ELK4 binding sites using qPCR. Relative enrichment was calculated over input DNA and plotted on the y axis. Each data point represents the average of triplicate ChIP experiments. Rabbit IgG was used as a non-specific control ChIP. Promoter regions tested for ChIP enrichment are shown on the x axis. The C-FOS promoter region is used as a positive control for ELK4 binding, CDH1 and CDH10 promoter regions were used as negative control regions for both, TR4 and ELK4 binding.

**Figure 10:**
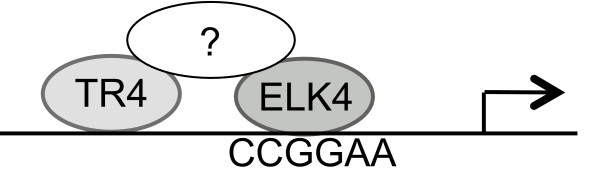
**Model of TR4-ELK4 *cis *module**. Gene promoters bound by both transcription factors, TR4 and ELK4, lack the DR1 element, but contain an ETS motif. This suggests that TR4 binding at these sites is facilitated through an ETS family member such as ELK4, possibly with the help of a bridging protein. TR4 may then augment ELK4 binding through non-specific DNA association, as depicted, or by serving as a non-DNA binding scaffold for additional accessory proteins.

To explore the possibility that ETS transcription factors might cooperate with TR4, we performed ChIP-seq analysis of ELK1 as well as ELK4 in HeLa cells and binding sites were determined using Sole-search. 2,312 ELK4 peaks were identified from 21 million reads and 702 ELK1 peaks were identified from 13 million reads, with 86% of the ELK1 sites also being ELK4 binding sites (see Additional file 4). When we compared the 1,135 TR4 targets present within 1 kb of a TSS with 1,715 ELK4 targets found within 1 kb of a TSS, a significant overlap of 30% was observed (Figure 8A; see Figure 9A for ChIP-seq binding pattern). To identify the motifs utilized for TR4 recruitment at the 346 TR4 binding sites that are also occupied by ELK4, we performed motif analysis using MEME. The ETS motif was highly overrepresented (E-value 3.3e-310), while the DR1 motif was not (E-value 2.5e + 4) (Figure 8B). We have thus identified a TR4-ELK4 *cis *module that accounts for 30% of TR4 binding sites. These sites are characterized by overrepresentation of the ETS motif in 96% of the sites and the lack of a DR1 element typically thought to recruit TR4. Therefore, TR4 does not directly bind to DNA via a DR1 element at these sites, but appears to be recruited through an ETS factor. We also analyzed the localization of binding relative to gene structure and found that TR4 and ELK4 display very similar patterns, with maximum binding between 500 bp upstream and downstream of a transcription start site (Figure 8C). The occurrence of both factors at common binding sites was confirmed by quantitative PCR using independent biological replicates (Figure 9B). Although we experimentally identified a *cis *regulatory module involving ELK4 at ~30% of TR4 binding sites, the ETS core motif was identified using bioinformatics to be within 53% of TR4 binding regions. It is possible that other ETS family members occupy these sites. It has been shown that the ETS family members ELK and GABPA shared half of their binding sites, while the other half were specific for a particular ETS factor [37]. Although further studies are needed, it is possible that ELK4 facilitates TR4 binding to promoter regions that do not contain the DR1 motif, suggesting the presence of ELK4 dependent and ELK4 independent modes of TR4 action (Figure 10).

## Conclusions

While it had been established that TR4 plays a critical role in embryonic development, differentiation and lipid metabolism, the modes by which it functions were previously unclear. To obtain a better understanding of the TR4 modes of action, we used ChIP-seq technology to identify TR4 target genes *in vivo *in multiple cell lines. This allowed us to confirm TR4 binding *in vivo *to the direct repeat of AGGTCA separated by one nucleotide (also known as a DR1 element) at endogenous target sites in all four cell types examined. Using *de novo *motif discovery, we found that the ETS motif CCGGAA was significantly overrepresented in TR4 binding sites, suggesting a role for ETS family members in TR4 action. To confirm the co-occurrence of these two factors *in vivo*, we performed ChIP-seq for the ETS transcription factor ELK4 and we found that about one third of TR4 target sites were indeed bound by ELK4. Sites that are bound by both factors contain an ETS motif, but lack the DR1 element typically thought to recruit TR4. These data suggest that TR4 may regulate specific subsets of target genes through ETS dependent as well as ETS independent pathways. Future studies will focus on the interdependence of these two transcription factors. Thus our approach of defining genome-wide binding patterns for a factor, followed by motif analysis to suggest possible *cis *modules, and then genome-wide analysis of the putative co-localizing factor has worked well to identify a TR4-ELK4 *cis *module.

Interestingly, we identified TR4 target genes that are common to quite diverse cell types (representatives of blood, liver, and epidermal cells). These genes were involved in fundamental biological processes such as RNA metabolism and protein translation. In addition, TR4 also binds near genes that are highly cell type-specific. For example, in HepG2 cells TR4 binds near genes that are involved in organic acid, lipid and carbohydrate metabolism. TR4 knockout mice show insulin hypersensitivity [16] and TR4 can be induced by certain essential fatty acids resulting in TR4 activation followed by the up-regulation of the apolipoprotein E precursor (ApoE) and cytosolic phosphoenolpyruvate carboxykinase 1 PEPCK gene [30], which is thought to contribute to diabetics-induced hyperglycemia [40,41]. Knowing the direct TR4 binding sites, it will be an interesting focus of future studies to evaluate the pathways underlying TR4 action and its possible role in metabolic diseases.

## Methods

### Cell culture and crosslinking

K562, HeLa, HepG2, and GM12878 cells for ChIP-seq were grown and crosslinked by the National Cell Culture Center (NCCC) as part of the ENCODE project. K562 and GM12878 cells were grown in RPMI supplemented with 10% fetal bovine serum (FBS), 2 mM L-Glutamine, 100 U/mL penicillin-streptomycin. HeLa and HepG2 cells were grown in DMEM medium supplemented with 10% FBS, 2 mM L-Glutamine, 100 U/mL penicillin-streptomycin. Cells were either processed for RNA isolation or crosslinked 10 minutes at a concentration of 1% formaldehyde, snap frozen and stored at -80C.

### Chromatin immunoprecipitation (ChIP) assay and library preparation

ChIP assays and the libraries for Illumina sequencing were prepared as described in detail in O'Geen et al. 2010 [42]. Briefly, chromatin from 10^8 ^cells was diluted with 5 volumes IP dilution buffer (50 mM Tris pH7.4, 150 mM NaCl, 1% (v/v) igepal, 0.25% (w/v) deoxycholic acid, 1 mM EDTA pH8) and incubated at 4C over night with either 50 μl of rabbit anti-TR4 antibody [15]. 300 μl protein A agarose beads were added for 2 hours to capture the immune complexes. Beads were washed three times with IP dilution buffer and once with phosphate-buffered saline. ChIP assays using 20 μl rabbit anti-ELK4 (Santa Cruz Biotechnology sc-13030X) or 20 μl of monoclonal rabbit anti-ELK1 (Epitomics #1277-1) were performed using StaphA cells as described on the Farnham lab web site (http://www.genomecenter.ucdavis.edu/farnham/pdf/FarnhamLabChIP%20Protocol.pdf). For sequencing experiments, StaphA cells were only blocked with BSA and the preclearing step was omitted. After reversal of crosslinks and RNase treatment, ChIP DNA was purified and used directly for library preparation.

### Sequencing and data analysis

Libraries were sequenced using the Illumina GA2 platform by the DNA Technologies Core Facility at the University of California-Davis (http://genomecenter.ucdavis.edu/dna_technologies/). The ChIP-seq data has been deposited in the NCBI Gene Expression Omnibus (accession number GSE24685). In addition, all TR4 ChIP-seq data can be visualized and downloaded from the UCSC browser at http://www.genome.ucsc.edu/cgi-bin/hgTrackUi?hgsid=169984430&c=chr9&g=wgEncodeYaleChIPseq. Peaks were called using the Sole-search software with default parameters (FDR0.0001, alpha value 0.001) using sequenced libraries of matched Input DNA for each cell type [21]. Peak overlap analysis based on chromosomal coordinates as well as location analysis were also performed using the Sole-search software. Gene Ontology analysis was performed using ConceptGen to identify the functional categories enriched in the overlapping targets in 4 cell types. (p-value < 0.05, modified Fisher's exact test). In addition to GO terms, other concepts were tested for significant enrichment in the gene set. All Entrez Genes were used as background to determine the significance of over-representation.

### Motif Analysis

*In vivo *binding sequences from TR4 peak files were retrieved from UCSC Genome Database (hg18, March 2006). Unbiased motif analysis was performed using MEME to identify statistically overrepresented motifs in the TR4 peak sequences present in 4 cell types. The following parameters were used "-dna -nmotifs 5 -mod zoops -minw 12 -maxw 20 -maxsize 2000000 -revcomp", which specify the number of motifs to search for, the zoops assumption (zero or one occurrence per peak sequence), the minimum motif length of 12 (length of a repeat element with no spacing between two half sites), the maximum motif length of 20 (length of a repeat element with 8 spacing nucleotides between two half sites), the maximum dataset size of 2,000,000 characters. Sequences were searched in forward and reverse orientation.

### RNA preparation and Illumina expression arrays

RNA was prepared from three independent cultures of 10^6 ^HeLa or HepG2 cells using Invitrogen Trizol according to the manufacture's recommendations. The Illumina TotalPrep RNA amplification kit from Ambion (AMIL1791) was used to generate biotinylated, amplified RNA for hybridization with the Illumina Sentrix Expression Beadchips, HumanHt-12. The Sentrix gene expression beadchips used for this study consisted of a 12-array, 2 stripe format comprising approximately 48 k probes/array. In this collection 24,000 probes were from RefSeq sequences and 24,000 from other Genbank sequences (see http://www.illumina.com/pages.ilmn?ID=197 for more details). Arrays were processed as per manufacturer's instructions, scanned at medium PMT settings as recommended by the manufacturer, and analyzed using Bead Studio Software v. 2.3.41. Data was normalized using the "average" method, which simply adjusts the intensities of two populations of gene expression values such that the means of the populations become equal. Relative expression values were calculated using an algorithm provided by Bead Studio. The expression array data has been deposited in the NCBI Gene Expression Omnibus (accession numbers GSE24419 for HepG2 and GSE19146 for HeLa data).

### ChIP assay and quantitative PCR (qPCR)

To confirm targets identified by ChIP-seq, all ChIP assays were performed using StaphA cells. 10^7 ^cells were used per ChIP experiment and adjusted amounts of the same antibodies and pre-immune serum (rabbit IgG) as described above. Immunoprecipitated DNA was purified and eluted in 50 μl water. 1 μl of ChIP DNA or 3 ng of Input DNA were used for qPCR analysis. Quantitative PCR experiments were performed at least in duplicates, from at least two independent ChIP assays on a Bio-Rad DNA Engine Opticon Real-Time PCR System using SYBR^® ^Green Master PCR Mix (SIGMA) according to the manufacturer's instructions. Results were analyzed relative to input. Each target site was calculated as 2 to the power of the cycle threshold (cT) difference between input DNA and ChIP samples. Enrichments at target sites are compared to negative/unbound control regions CDH1 and CDH10 (see Additional file 2 for primer sequences).

## Authors' contributions

HOG designed, performed and analyzed experiments and drafted the manuscript. YHL performed bioinformatics analysis, provided figures and text for the manuscript. XX performed ChIP-seq experiments and data analysis. LE performed ChIP-seq experiments and data analysis. VMK helped with RNA and ChIP-seq data analysis and provided figures and text for the manuscript. DH performed QPCR experiments and data analysis. SF performed RNA experiments and data analysis. OT and LS generated and purified anti-TR4 antisera. MAS assisted with bioinformatics analysis. JDE and PJF helped with experimental design and writing of manuscript. All authors read and approved the final manuscript.

## Supplementary Material

Additional file 1 **Validation of TR4 expression in four different cell types**. Western blot analysis to validate expression of TR4 protein. 10 μg nuclear extract were loaded per lane. Cell types used are indicated above each lane. Click here for file

Additional file 2 **Primer sequences for standard and qPCR validation of TR4 ChIP samples**. Click here for file

Additional file 3 **PCR anaslysis of three TR4 binding sites**. ChIP assays were performed in K562 and HepG2 cells using TR4 antibody. PCR was performed using primers to TR4 binding sites identified by ChIP-seq (see Additional file 2 for oligo sequences). The enrichment of TR4 is shown in comparison to 0.1% Input chromatin. IgG ChIPs were used as negative controls. PCR analysis confirmed presence of TR4 at TNFIAP1, ECSIT and SCAP, but showed no significant enrichment when using negative control primers to ZNF333. Click here for file

Additional file 4 ChIP-seq data and analysis summary Click here for file

## References

[B1] Vaquerizas JM Kummerfeld SK Teichmann SA Luscombe NM A census of human transcription factors: function, expression and evolution Nat Reviews Genetics 2009 10 252 263 10.1038/nrg2538 19274049

[B2] Farnham PJ Insights from genomic profiling of transcription factors Nature Rev Genet 2009 605 616 10.1038/nrg2636 19668247 PMC2846386

[B3] Park PJ ChIP-seq: advantages and challenges of a maturing technology Nature Rev Genet 2009 669 680 10.1038/nrg2641 19736561 PMC3191340

[B4] Mangelsdorf DJ Thummel C Beato M Herrlich P Schutz G Umesono K Blumberg B Kastner P Mark M Chambon P Evans RM The nuclear receptor superfamily: the second decade Cell 1995 83 835 839 10.1016/0092-8674(95)90199-X 8521507 PMC6159888

[B5] Sandelin A Wasserman WW Prediction of nuclear hormone receptor response elements Mol Endocrinol 2005 19 595 606 10.1210/me.2004-0101 15563547

[B6] Noy N Ligand specificity of nuclear hormone receptors: sifting through promiscuity Biochemistry 2007 46 13461 13467 10.1021/bi7018699 17983246

[B7] Lee YF Lee HJ Chang C Recent advances in the TR2 and TR4 orphan receptors of the nuclear receptor superfamily J Steroid Biochem Mol Biol 2002 81 291 308 10.1016/S0960-0760(02)00118-8 12361719

[B8] Su Liu SX Lee Yi-fen Chang Chawnshang Physiological Functions of TR2 and TR4 Orphan Nuclear Receptor 2010 Nuclear Receptors: Current Concepts and Future Challenges: Springer Netherlands 327 343

[B9] Chang C Da Silva SL Ideta R Lee Y Yeh S Burbach JP Human and rat TR4 orphan receptors specify a subclass of the steroid receptor superfamily Proc Natl Acad Sci USA 1994 91 6040 6044 10.1073/pnas.91.13.6040 8016112 PMC44133

[B10] Bookout AL Jeong Y Downes M Yu RT Evans RM Mangelsdorf DJ Anatomical profiling of nuclear receptor expression reveals a hierarchical transcriptional network Cell 2006 126 789 799 10.1016/j.cell.2006.06.049 16923397 PMC6211849

[B11] Lee YF Pan HJ Burbach JP Morkin E Chang C Identification of direct repeat 4 as a positive regulatory element for the human TR4 orphan receptor. A modulator for the thyroid hormone target genes J Biol Chem 1997 272 12215 12220 10.1074/jbc.272.18.12215 9115296

[B12] Tanabe O Shen Y Liu Q Campbell AD Kuroha T Yamamoto M Engel JD The TR2 and TR4 orphan nuclear receptors repress Gata1 transcription Genes Dev 2007 21 2832 2844 10.1101/gad.1593307 17974920 PMC2045135

[B13] Tanabe O Katsuoka F Campbell AD Song W Yamamoto M Tanimoto K Engel JD An embryonic/fetal beta-type globin gene repressor contains a nuclear receptor TR2/TR4 heterodimer EMBO J 2002 21 3434 3442 10.1093/emboj/cdf340 12093744 PMC126089

[B14] Omori A Tanabe O Engel JD Fukamizu A Tanimoto K Adult stage gamma-globin silencing is mediated by a promoter direct repeat element Mol Cell Biol 2005 25 3443 3451 10.1128/MCB.25.9.3443-3451.2005 15831451 PMC1084292

[B15] Tanabe O McPhee D Kobayashi S Shen Y Brandt W Jiang X Campbell AD Chen YT Chang C Yamamoto M Tanimoto K Engel JD Embryonic and fetal beta-globin gene repression by the orphan nuclear receptors, TR2 and TR4 EMBO J 2007 26 2295 2306 10.1038/sj.emboj.7601676 17431400 PMC1864974

[B16] Liu NC Lin WJ Kim E Collins LL Lin HY Yu IC Sparks JD Chen LM Lee YF Chang C Loss of TR4 orphan nuclear receptor reduces phosphoenolpyruvate carboxykinase-mediated gluconeogenesis Diabetes 2007 56 2901 2909 10.2337/db07-0359 17827404

[B17] Kim E Yang Z Liu NC Chang C Induction of apolipoprotein E expression by TR4 orphan nuclear receptor via 5' proximal promoter region Biochem Biophys Res Commun 2005 328 85 90 10.1016/j.bbrc.2004.12.146 15670754

[B18] Chen LM Wang RS Lee YF Liu NC Chang YJ Wu CC Xie S Hung YC Chang C Subfertility with defective folliculogenesis in female mice lacking testicular orphan nuclear receptor 4 Mol Endocrinol 2008 22 858 867 10.1210/me.2007-0181 18174360 PMC2725750

[B19] Shyr CR Kang HY Tsai MY Liu NC Ku PY Huang KE Chang C Roles of testicular orphan nuclear receptors 2 and 4 in early embryonic development and embryonic stem cells Endocrinology 2009 150 2454 2462 10.1210/en.2008-1165 19131575

[B20] Xie S Lee YF Kim E Chen LM Ni J Fang LY Liu S Lin SJ Abe J Berk B Ho FM Chang C TR4 nuclear receptor functions as a fatty acid sensor to modulate CD36 expression and foam cell formation Proc Natl Acad Sci USA 2009 106 13353 13358 10.1073/pnas.0905724106 19666541 PMC2726407

[B21] Blahnik KR Dou L O'Geen H McPhillips T Xu X Cao AR Iyengar S Nicolet CM Ludaescher B Korf I Farnham PJ Sole-search: An integrated analysis program for peak detection and functional annotation using ChIP-seq data Nucleic Acids Res 2010 38 e13 10.1093/nar/gkp1012 19906703 PMC2817454

[B22] Xu X Bieda M Jin VX Rabinovich A Oberley MJ Green R Farnham PJ A comprehensive ChIP-chip analysis of E2F1, E2F4, and E2F6 in normal and tumor cells reveals iterchangeable roles of E2F family members Genome Res 2007 17 1550 1561 10.1101/gr.6783507 17908821 PMC2045138

[B23] Fry CJ Pearson A Malinowski E Bartley SM Greenblatt J Farnham PJ Activation of the murine dihydrofolate reductase promoter by E2F1: A requirement for CBP recruitment J Biol Chem 1999 274 15883 15891 10.1074/jbc.274.22.15883 10336493

[B24] Fujiwara T O'Geen H Keles S Blahnik K Linnemann AK Kang YA Choi K Farnham PJ Bresnick EH Discovering hematopoietic mechanisms through genome-wide analysis of GATA factor chromatin occupancy Mol Cell 2009 36 667 681 10.1016/j.molcel.2009.11.001 19941826 PMC2784893

[B25] Sartor MA Mahavisno V Keshamouni VG Cavalcoli J Wright Z Karnovsky A Kuick R Jagadish HV Mirel B Weymouth T Athey B Omenn GS ConceptGen: a gene set enrichment and gene set relation mapping tool Bioinformatics 26 456 463 10.1093/bioinformatics/btp683 20007254 PMC2852214

[B26] Tsai NP Huq M Gupta P Yamamoto K Kagechika H Wei LN Activation of testicular orphan receptor 4 by fatty acids Biochim Biophys Acta 2009 1789 734 740 19800043 10.1016/j.bbagrm.2009.09.010 PMC2784022

[B27] Lee CH Chinpaisal C Wei LN A novel nuclear receptor heterodimerization pathway mediated by orphan receptors TR2 and TR4 J Biol Chem 1998 273 25209 25215 10.1074/jbc.273.39.25209 9737983

[B28] Ravasi T Suzuki H Cannistraci CV Katayama S Bajic VB Tan K Akalin A Schmeier S Kanamori-Katayama M Bertin N Carninci P Daub CO Forrest AR Gough J Grimmond S Han JH Hashimoto T Hide W Hofmann O Kamburov A Kaur M Kawaji H Kubosaki A Lassmann T van Nimwegen E MacPherson CR Ogawa C Radovanovic A Schwartz A Teasdale RD An atlas of combinatorial transcriptional regulation in mouse and man Cell 2010 140 744 752 10.1016/j.cell.2010.01.044 20211142 PMC2836267

[B29] White R Morganstein D Christian M Seth A Herzog B Parker MG Role of RIP140 in metabolic tissues: connections to disease FEBS Lett 2008 582 39 45 10.1016/j.febslet.2007.11.017 18023280

[B30] Huq MD Gupta P Tsai NP Wei LN Modulation of testicular receptor 4 activity by mitogen-activated protein kinase-mediated phosphorylation Mol Cell Proteomics 2006 5 2072 2082 10.1074/mcp.M600180-MCP200 16887930

[B31] Lee W Tillo D Bray N Morse RH Davis RW Hughes TR Nislow C A high-resolution atlas of nucleosome occupancy in yeast Nat Genet 2007 39 1235 1244 10.1038/ng2117 17873876

[B32] Lee YF Young WJ Burbach JP Chang C Negative feedback control of the retinoid-retinoic acid/retinoid X receptor pathway by the human TR4 orphan receptor, a member of the steroid receptor superfamily J Biol Chem 1998 273 13437 13443 10.1074/jbc.273.22.13437 9593676

[B33] Jin V Rabinvich A Squazzo SL Green R Farnham PJ A computational genomics approach to identify cis-regulatory modules from chromatin immunoprecipitation microarray data-a case study using E2F1 Genome Research 2006 16 1585 1595 10.1101/gr.5520206 17053090 PMC1665642

[B34] FitzGerald PC Shlyakhtenko A Mir AA Vinson C Clustering of DNA sequences in human promoters Genome Res 2004 14 1562 1574 10.1101/gr.1953904 15256515 PMC509265

[B35] Jin V Apostolos J Nagisetty NS Farnham PJ W-ChIPMotifs: a web application tool for de novo motif discovery from ChIP-based high throughput data Bioinformatics 2009 25 3191 3193 10.1093/bioinformatics/btp570 19797408 PMC2778340

[B36] Valouev A Johnson DS Sundquist A Medina C Anton E Batzoglou S Myers RM Sidow A Genome-wide analysis of transcription factor binding sites based on ChIP-seq data Nature Methods 2008 5 829 834 10.1038/nmeth.1246 19160518 PMC2917543

[B37] Boros J Donaldson IJ O'Donnell A Odrowaz ZA Zeef L Lupien M Meyer CA Liu XS Brown M Sharrocks AD Elucidation of the ELK1 target gene network reveals a role in the coordinate regulation of core components of the gene regulation machinery Genome Res 2009 19 1963 1973 10.1101/gr.093047.109 19687146 PMC2775591

[B38] Boros J O'Donnell A Donaldson IJ Kasza A Zeef L Sharrocks AD Overlapping promoter targeting by Elk-1 and other divergent ETS-domain transcription factor family members Nucleic Acids Res 2009 37 7368 7380 10.1093/nar/gkp804 19789270 PMC2794171

[B39] O'Donnell A Yang SH Sharrocks AD MAP kinase-mediated c-fos regulation relies on a histone acetylation relay switch Mol Cell 2008 29 780 785 18374651 10.1016/j.molcel.2008.01.019 PMC3574235

[B40] Gomez-Valades AG Vidal-Alabro A Molas M Boada J Bermudez J Bartrons R Perales JC Overcoming diabetes-induced hyperglycemia through inhibition of hepatic phosphoenolpyruvate carboxykinase (GTP) with RNAi Mol Ther 2006 13 401 410 10.1016/j.ymthe.2005.08.026 16271515

[B41] Valera A Pujol A Pelegrin M Bosch F Transgenic mice overexpressing phosphoenolpyruvate carboxykinase develop non-insulin-dependent diabetes mellitus Proc Natl Acad Sci USA 1994 91 9151 9154 10.1073/pnas.91.19.9151 8090784 PMC44765

[B42] O'Geen H Frietze S Farnham PJ Using ChIP-seq technology to identify targets of zinc finger transcription factors Methods Mol Biol 2010 649 437 455 20680851 10.1007/978-1-60761-753-2_27 PMC4151297

